# U.S. Paralympic Hopeful's Athletic Identity and How It Has Been Affected by the Sport Disruption of COVID-19

**DOI:** 10.3389/fspor.2021.689555

**Published:** 2021-07-20

**Authors:** Tiao Hu, Mathew Mendoza, Joy Viray Cabador, Michael Cottingham

**Affiliations:** Department of Health and Human Performance, Lab of Adaptive Athletics at University of Houston, University of Houston, Houston, TX, United States

**Keywords:** athletic identity, paralympian, sport disruption, COVID-19 pandemic, disability sport, Tokyo 2020 olympic and paralympic games, sport psychology, paralympics

## Abstract

The purpose of this study was to explore the status of Paralympic hopefuls' athletic identity and how this identity was impacted by the training and competition cessation resulting from the COVID-19 pandemic. Researchers conducted in-depth semi-structured interviews that explored the experiences of 29 Paralympic hopefuls who compete in thirteen different Paralympic sports. A thematic analysis yielded two superordinate themes: a) Prominent athletic identity, multiplicity over exclusivity; b) Various Impact on AI: Mental adaptation helps overcome the lack of sport participation. Participants in this study possessed prominent strong athletic identities from the benefits of sport participation. Their prioritized athletic role still remains despite setbacks due to the pandemic. However, athletes identified with multiple roles rather than an exclusive athletic identity during COVID-19. As for the impacts on identity, the severity of challenges are determined by the mindset of the athletes. All of the athletes experienced a decreased amount of time and physical participation in their sport. Paralympians whose sole focus was on the loss of physical participation were impacted the most. Athletes who felt unchallenged did so because of their mental adaptation. Through a positive outlook and mentality, athletes were able to effectively cope and not dwell on the negative aspects brought on by the pandemic. In conclusion, having a strong AI did not necessarily coincide with a negative impact on identity from COVID-19, and those who do not possess a strong AI felt their AI was unchallenged by the pandemic. More importantly, Paralympians' mindset of how they view and interpret their AI is crucial to how the individual's AI is affected by the sport disruption of COVID-19.

## Introduction

The ongoing coronavirus disease (COVID-19) caused a worldwide pandemic, most strongly recognized during its surge in 2020, when all communities and societies were forced to adopt drastic changes to curb its spread. In the sport realm, an announcement made Mar. 24th, 2020 declared that the Tokyo 2020 Olympic and Paralympic Games would be postponed for 1 year, causing great disruption among athletes who had trained long and hard for years to participate. Suddenly, athletes experienced lockdown, travel bans, tournament cancelations, facility shutdowns, social distancing, as well as other governmental guidelines and safety protocols—botching the athletes' competitive calendars and routines. This undoubtedly presented challenges as they had limited to no access to effective training environments, partners and teammates (Schinke et al., 2020).

Several researchers have investigated the impact that such inactivity conditions may have on physiological systems, as well as on athletic performance (Jukic et al., 2020). However, the effects of the global pandemic on athletes' Athletic Identity, an important component of athlete's self-concept and health and performance outcomes, is still to be explored. Athletic identity (AI) was examined based on sport-related benefits (i.e., physical self-efficacy, enhanced body image), and with the premise that it would be manifested most strongly in athletes whose self-concepts were significantly tied into the athlete role (Martin, 1999). Although developing a strong athletic identity can be salient and beneficial for active athletes, athletes with too exclusive of an AI may have emotional difficulty adjusting to non-sport participation (Werthner and Orlick, 1986).

Scant research has only begun to track AI changes among non-disabled athletes during the COVID-19 pandemic, which forced a decline in sport participation. The recent knowledge reveals that during the lockdown period, elite athletes, and team sports athletes showed higher AI compared to other level and individual sport athletes; however, athletes with higher AI tend to ruminate and catastrophize more (Costa et al., 2020). Student-athletes did not uniformly experience identity loss following school closures; some of them even reported stronger AI at a certain time mainly due to maintaining social interaction and team support (Graupensperger et al., 2020).

Currently, there is no identified research exploring the status of the athletes with disabilities' AI and how it is impacted by COVID-19. We will argue that Paralympic-level athletes who were training for the pinnacle sporting event of their careers were uniquely situated, in that their AI may be influenced by the new challenges brought on by the pandemic. Paralympic-level athletes already face challenges including overtraining and injury, normalized pain, and health hazards (Fagher et al., 2016). Moreover, they continue to struggle with external barriers such as lack of sufficient adaptive sport facilities, as well as logistical hurdles in travel to competition sites (Swartz et al., 2019).

With the uncertainty of when it will be safe to return to athletic participation, Paralympians are now up against new straining circumstances due to COVID-19's impact on their physical, social, and even financial state. They rely very heavily on the Games, as it represents the greatest chance for single-source income for most Paralympians in the U.S. For those who have side jobs to support their livelihoods, the pandemic may still cause financial hardships resulting from job or sponsorship loss (Taku and Arai, 2020). Based on the aforementioned considerations, the aim of the current study was to examine the status of Paralympic hopefuls' athletic identity and how this identity was impacted by the training and competition cessation resulting from the COVID-19 pandemic.

## Literature Review

### Athletic Identity

Athletic identity (AI) is based in social role theory and refers to the degree with which an individual identifies with the athletic role (Brewer et al., 1993). It is considered both a cognitive structure (schema) and a social role. AI is known to be a pervasive and important factor for health and fitness outcomes, global self-esteem, social relationships, and commitment to sport and physical activity (Horton and Mack, 2000). Furthermore, it effectively creates positive impressions regardless of sex, age, or activity level, as well as able-bodiedness (Hutzter and Bar-Eli, 1993; Manns and Chad, 1999).

People with strong AI have self-schema grounded in being an athlete. Possessing a strong AI can have both positive and negative effects. Athletes who possessed a stronger AI were more committed to their sport and had better athletic performances (Horton and Mack, 2000); however, they were more likely to neglect other parts of their lives to fulfill their athletic role (Cornelius, 1995; Murphy et al., 1996) and were at greater risk of emotional and psychological distress while adjusting upon injury and retirement (Brewer, 1993; Grove et al., 1997; Webb et al., 1998).

Strength of athletic identity is thought to vary with past and current athletic experience and the relative success or failure in this domain (Horton and Mack, 2000); it may be assessed through the 3-factor structure (social identity, exclusivity, and negative affectivity) Athletic Identity Measurement Scale (AIMS) (Brewer et al., 1993). While we have a good understanding on the conceptualization of AI, very few studies examined how AI was maintained, managed, and changed. Through Poucher and Tamminen (2017), we know elite athletes appeared to maintain AI through personal actions and behaviors (e.g., compartmentalization, justified commitment) as well as environmental factors (e.g., attention from others, daily routines).

### Disability Sport Athletic Identity

Regarding disability sport AI, the present body of literature follow two distinct research lines: Quantitative research examining potential antecedents, correlates, and outcomes of the AI, and qualitative research learning how athletes feel about being an athlete while having a disability (Guerrero and Martin, 2018). By using in-depth interviews and focusing on personal narratives, qualitative studies capture the broad and complex dynamics of identity construction, development, and negotiation process (Martin, 2013).

How AI is constructed within populations of athletes with disabilities is still relatively unknown. The only identified research on athletes with intellectual disabilities shows that participants generally do not identify themselves with the role of a sportsman (Wilinski et al., 2014). Correspondingly, research investigating the influence of sport on athletes with physical disabilities' sense of self and identity development is also considered limited (Pack et al., 2017). What is known, is that sport as a domain has been identified as a venue that produces favorable self-perceptions among individuals with disabilities (Arbour et al., 2007; Giacobbi et al., 2008; Ginis et al., 2010), while also facilitating AI development (Anderson, 2009) and AI strengthening (Ioannis et al., 2018). Disability sport participation both predicts and results in AI development (Groff and Zabriskie, 2006; Tasiemski and Brewer, 2011; Perrier et al., 2012), meaning that the relationship between sport participation and AI is likely bidirectional and thus verifies the value of sport participation for people with disabilities.

Indeed, involvement in sports and AI development could potentially help people with disabilities to repair and redefine themselves, combating social marginalization. In doing so, they are recognized as “real” athletes by themselves and by society, are able to cope efficiently with the disability state (Wheeler et al., 1999; Swartz et al., 2018); they transform their disability identity to that of an elite athlete (Le Clair, 2011) and enhance their well-being (Nagata, 2014). These athletes who possess an affirmative athlete identity are proud to be disabled and experience benefits of living with a disability. This so-called affirmative model (Swain and French, 2000), which focuses on positive experiences and social identities for people with disabilities. It explains athletes with disabilities who embrace a disability sport AI during the identity negotiation process, compare to those reject it, and those who will not likely develop an AI (Sparkes and Smith, 2002). Thus, a strong AI is correlative with aspects of personal and social identities.

A strong disability sport AI is also related to various psychological factors, such as perceived competence (Shapiro, 2003), quality of life (Groff et al., 2009), self-esteem (Marin-Urquiza et al., 2018), and physical self-confidence (Van de Vliet et al., 2008). Several other factors have been linked to disability sport AI formation: athletes with disabilities' profile factors like family status, level of education, disability, athletic experience, and type of games (Ioannis et al., 2018), nationality differences (Groff and Zabriskie, 2006), and the athletes' role during competition and their type of sport (Tasiemski and Brewer, 2011). Further, Anderson (2009) found that factors like confidence in sporting abilities, a strong commitment to sport goals, a sense of community, and significant others' support are important elements that facilitated the development of disability sport AI.

There is a divergence of the findings in previous research regarding relationship between AI and negative affectivity, life satisfaction, and sport performance (Tasiemski et al., 2004, 2013; Wiśniowska et al., 2012). However, to date, research has demonstrated equally strong AI between athletes with disabilities when compared to those without a disability (Groff and Zabriskie, 2006). Researchers confirmed that athletes with disabilities strongly considered themselves as athletes (Ioannis et al., 2018), and have many sport-related goals as well as a strong desire to achieve these goals (Martin et al., 1995).

In contrast with non-disabled AI, there is conflicting evidence that the 3-factor structure is maintained when the AIMS is used among athletes with disabilities (Martin et al., 1997; Groff and Zabriskie, 2006; Ioannis et al., 2018). Despite ongoing controversy in regards to the best use of the measurement scale, we know disability sport AI tends to be stronger among men (Brewer and Cornelius, 2001), older athletes (Brewer et al., 1993), and elite athletes (Tasiemski et al., 2004). Strength of AI also increases with the number of hours devoted to practice (Tasiemski and Brewer, 2011), experience, accomplishments, and competence (Le Clair, 2011).

A disability sport AI does not end when sport ends. Given that athletes with disabilities' career has been cited as a central part of an athlete's identity (Wheeler et al., 1999), the sport career transition (e.g., retirement) received comparable attention. Research showed that athletes with disabilities who were retired and considering retiring had lower AI and poorer performance compared with those who did not plan to retire, and no relationship was found between perceived stress and AI (Martin and Ridler, 2014). Likewise, athletes with disabilities experience the same negative psychological outcomes of AI, such as post-injury depression (Buman et al., 2008) and difficulties transitioning to retirement (Grove et al., 1997; Marin-Urquiza et al., 2018).

With the aforementioned heavy attention in disability sport AI, it is worth noting that the self-schemas of many athletes with disabilities are not limited solely to their AI and an athlete may describe oneself through multiple identities (Martin, 1999; Huang and Brittain, 2006). It is in line with the concept of multiplicity in personality psychology research, which refers to people who identify as if they have at least two distinct selves (Spanos, 1994). To further understand AI in the disability sport context, an important yet rarely investigated group, Paralympic athletes, were chosen as representatives for this study.

### Athletic Identity of Paralympians

The Paralympic Games is a quadrennial global multi-sport competition for individuals with certain impairments. This elite sport event has become increasingly competitive and larger over the years, which consequently resulted in heightened expectations of participating para-athletes with regards to time spent training and the achievement of podium performances (Hammond and Jeanes, 2018). The anticipated highest level of competition and considerable sporting achievement leads to the recognition of “being an athlete” and is associated with Paralympian's AI.

Few researchers have examined Paralympians' AI. Quantitative research has focused on relationships between Paralympians' AI score, sport performance, self-perception, and retirement status. Paralympic athletes report strong AI, self-esteem and physical self-perceptions compared to non-disabled sportive individuals. Paralympians' AI levels were relatively stable compared with the non-Paralympians over time. However, no relationship was found between AI and (physical) self-perceptions (Van de Vliet et al., 2008). Further, active Paralympians have a stronger AI and better performance compared to those who are retired or are considering retirement. Athletes who have a higher scores of AI, exclusivity and negative affectivity would likely to experience a higher degree of depressive symptoms upon retirement. Also, no correlation has been found between AI, retirement status (active, retired), sport performance with self-esteem (Tasiemski et al., 2013; Martin and Ridler, 2014; Marin-Urquiza et al., 2018).

Qualitative research focuses on Paralympians' identity development through learning how Paralympic athletes described their identities and how they meshed perceptions of sport, impairment, disability, and life. Research has shown that sport participation enhanced self and social acceptance, identity development, and quality of life. Paralympians did not view themselves as having lost something, as being disabled, nor as supercrips, which support the Affirmation Model of Disability (Pack et al., 2017; Swartz et al., 2019). In contrast, Huang and Brittain (2006) reported that most Paralympians assigned their impairment as their “master identity status” instead of AI, which follows the medical model. This study also briefly commented on culture and identity as broader influences on identity development. Moreover, it highlights how being a Paralympian uniquely contributed to the transformation process of identity development by experiencing success through international disability sport.

Meanwhile, Le Clair (2011) also highlighted that the elite athlete status of being a Paralympian contributes to an identity transformation from a disability identity to an AI. This AI development results from the experience of accomplishment and competence in professional sport. Moreover, the study emphasized the unique platform of the international Paralympic Games and its institutional transformation from a disability rehabilitation-based organization to an elite sport-based organization. This shift contributes to the transformation of the Paralympians' identity.

We understand that identities are constantly in the process of change and transformation, and Paralympians have multiple identities outside of their AI, and these identities shift over time as athletes go through life changes (Huang and Brittain, 2006). Significant life events relating to sport disruption (e.g., retirement, parenthood) are often catalysts for identity changes and challenges. Paralympians—who possibly possess the strongest disability sport AI—face unique identity challenges related to sport disruption.

### Sport Disruptions and Athletic Identity

To better understand the possible effects of COVID-19 on athlete identity, we examined sport disruptions, the closest identified analog. Career transitions brought on by athletic disturbances can result in a negative impact on AI (Park et al., 2012). These athletic disturbances typically lead to a partial or complete stop of sport participation and include, injury, retirement, and parenthood (Kerr and Dacyshyn, 2000; Tekavc et al., 2020; Caron et al., 2021). While the latter has a contrasting effect, sport disruptions such as injury and retirement can be detrimental to elite athletes' psychology which ultimately impairs their AI. This is a result of the sometimes-lengthy hiatus interfering with their normal sport participation. Because sport so deeply influences an elite athletes' way of living and manner of socialization, injury affects athletes' physical health as well as mental health (Caron et al., 2017). The mental health impacts that often arise accompanying these life-altering events, ranging from feeling neglected by their support systems, to struggling to figure out self-identity (Caron et al., 2021). Some elite athletes describe their AI struggles from sport disruption as being in “Nowhere Land”- feeling an overwhelming sense of identity confusion and life uncertainty (Kerr and Dacyshyn, 2000). They may even lose characteristics of their personality (Blinde and Stratta, 1992; Muscat, 2010). Experiencing severe depression and a loss of status and self-confidence, athletes' feel inferior to their previous self and former teammates. In addition to the psychological stressors, sport disruptions can have physical impacts on the athlete including sleep disorders, appetite loss, and alcohol abuse (Blinde and Stratta, 1992; Sinclair and Orlick, 1993).

It is important to note that though these negative effects from sport disruption are significant and well-studied, it has also been found that they can be precluded if athletes proactively retract themselves from sport and take initiative to develop identity in other areas. Research has concluded that the application of identity multiplicity is a significant factor in preventing AI difficulty for these athletes (Lally, 2007; Muscat, 2010). In addition, a particular sport disruption, motherhood, has been shown to self-enlighten athletes. Athlete mothers are able to discover different self-dimensions which ultimately decrease sport competitive pressure (Appleby and Fisher, 2009). This is beneficial for athlete's mentality when they return to sport, as they described having a stronger motivation to train, a stronger wish to compete in tournaments, and the desire to make their performance at the Olympic Games a family experience (Tekavc et al., 2020).

Throughout this body of literature, it is apparent that there are negative effects on identity from sport disruption when elite athletes do not make an effort to identify in other life domains. Further, it is acknowledged that a need for counseling exists to assist elite athletes in adjusting to life without sport. The discussed information correlates with the experiences of non-disabled elite athletes, as many studies have focused on this population when studying sport disruption. In contrast, however, studies on elite disability sport and populations of Paralympic athletes is lacking.

Limited studies in the disability sport context have only briefly touched on how the disruptions of injury and retirement can be a psychological stressor, resulting in negative feelings such as depression and anxiety for Paralympians (Fagher et al., 2016; Marin-Urquiza et al., 2018). Further, there are experiences from sport disruptions that are unique to elite athletes with disabilities that need to be explored more in depth. These experiences include retirement from declassification. Declassification in para-sports involves a system of segmenting, and in some cases excluding, athletes based on the type of impairment and whether they meet the “Minimum Impairment Criteria”- regardless of their performance level. For this reason, there is no able-body equivalent (Bundon et al., 2018). Yet, while it has been found that Paralympians' AI is negatively affected by retirement, the area of sport disruption in general on AI of Paralympians is greatly lacking. Research in this field can help explain the causes and solutions to athlete's psychological traumas, financial struggles, and illness' that coincide with AI difficulties.

A unique and collective sport disruption that all, if not most, Paralympians have experienced starting in 2020 is the ongoing COVID-19 pandemic. By investigating how COVID-19 has affected the AI of Paralympians, we hope to add to this body of literature, as well as discuss the unique challenges para-athletes specifically face. Studying this will aid in understanding Paralympians' AI and how it is affected by sport disruptions. The purpose of our study is to examine the status of Paralympic hopefuls' athletic identity and if that identity has been impacted from the training and competition cessation caused by the COVID-19 pandemic.

## Methods

### Design of Study

Researchers had increasingly called for a qualitative manner to better understand the unique yet complex process of athletes with disabilities' identity development. Moreover, with limited previous research regarding Paralympian's AI and no equivalent sport disruption event like COVID-19, we deem it is important to track changes of Paralympian's AI based on acknowledging their AI status. Thus, as the first attempt seeking to understand how participating in sport and Paralympic Games would result in developing a disability sport AI and how it would be impacted by COVID-19, we employed an exploratory qualitative methodology. To achieve the research goal, two research questions were addressed through this study:

RQ1: What is Paralympic hopeful's athletic identity?
RQ2: Have Paralympic Hopefuls' athletic identity been impacted by COVID-19; if so, how?

### Procedures

All research assistants that collected data worked in an established disability sport lab and had been exposed to disability sports for at least 6 months to a year. These individuals took part in leadership roles running disability sport programming. Following, the individuals transitioned into conducting research where they were trained in qualitative research and data collection. The project was led by a tenured senior faculty member who has conducted qualitative research and has a number of peer-reviewed publications. The training process included multiple observations of interviews, practice interviews, partnering in observing and listening to practice interviews, gaining feedback through the practice interview process, and being trained in asking follow-up questions.

All individuals were trained in qualitative data collection and engaged in bracketing in order to address issues of reliability (Tufford and Newman, 2012). Bracketing was used to mitigate biases and preconceptions of personal experience, interest, values, and emotions. This is significant in being aware of one's preconceptions, as it influences the research assistants perceptions toward a subject or their interpretation of a subject's response (Darawsheh, 2014). The specific process of bracketing was to individually self-reflect and list out every bias or influence that could have impacted data collection or interpretation of the findings. Following this, every individual was instructed to share their form of bracketing to a close family member or friend to challenge validity of the biases and to further reflect on the influences that can cause predetermined disposition.

A reflection of roles in the context of what it means to work with a person with a disability was discussed prior with the senior faculty member of this study. While the senior faculty member on this study does have a physical disability, none of the other individuals presented a disability. However, it should be acknowledged that world views and experiences will be different from the participants who did have physical disabilities. Researchers were also trained to ethically engage with the athletes throughout interviews, as they are freely and voluntarily participating in the research (Seidman, 2006).

A total of five research assistants participated in data collection, consisting of two males and three females, ages ranging from 20 to 25 years old, with ethnicities including Hispanic, Middle Eastern, Asian, and African American. Out of the five research assistants, two individuals that were involved in data collection furthered the data analysis and continued to be authors. An additional author was added for data analysis and to further the study for publication.

Data was managed in a way that protected confidentiality by providing pseudonyms for all subjects. The engagement in practice interviews provided research assistants the experience to effectively probe for responses while still respecting the participants' privacy and desire to discuss or not discuss certain topics, especially questions that might be sensitive to them.

### Participants and Participant Selection

Purposeful sampling was used for selection of participants. Paralympic Hopefuls who were qualified or attempting to qualify for the 2020 Paralympics were recruited for the study. Athletes meeting the criteria were invited by the research assistants to participate in the study. Coaches and administrators were contacted via email, posting on social media, and using snowball sampling. Athletes who wanted to participate in the study contacted the lab via email. Following, a consent form was emailed to the athletes. After the athletes reviewed the consent form, they were instructed to give a verbal or digital confirmation that they accept and agree to the study. Once approved, a date and time was scheduled between the athlete and two research assistants for an interview. Every athlete that participated in the interview process received a $25 gift card that was provided by the Lab of (name redacted until article accepted). Twenty-nine Paralympic hopefuls, ages ranging from 18 to 62 participated in this study. Table 1 provides a detailed description of our sample, including pseudonym, gender, specific sport participation, and nature of disability. The results section gives a broader picture of who the athletes are and how sport has influenced their current identity as a Paralympic Hopeful who self identifies as an athlete.

**Table 1:** Demographic information of athletes.

**Pseudonyms**	**Gender**	**Sport**	**Nature of disability**
Cooper	Male	Boccia	Cerebral Palsy
Neal	Male	Cycling	Cerebral Palsy
Stephanie	Female	Equestrian	Wyburn Mason Syndrome
Katherine	Female	Equestrian	Brain Stem Cerebellar Injury
Marge	Female	Equestrian	Multiple Sclerosis
Trisha	Female	Fencing	Primary Cerebellar Degeneration
Valerie	Female	Fencing	Ehlers-Danlos Syndrome
Connor	Male	Goalball	Retinitis Pigmentosa
Abbie	Female	Goalball	Visual Impairment
Maranda	Female	Goalball	Visual Impairment
Leslie	Female	Goalball	Visual Impairment
Allie	Female	Goalball	Congenital Glaucoma
Kendall	Female	Paracanoeing	Spinal Cord Injury
Cara	Female	Paracanoeing	Spinal Cord Injury
Campbell	Male	Powerlifting	Spina Bifida
John	Male	Powerlifting	Amputee
Jacob	Male	Rugby	Spinal Cord Injury
Natalie	Female	Sitting Volleyball	Umbilical Band Syndrome
Brittney	Female	Sitting Volleyball	Fibular Hemimelia
Lilly	Female	Sitting Volleyball	Amputee
Michelle	Female	Swimming	Visual Impairment
Stewart	Male	Swimming	Neuroblastoma
Brandy	Female	Taekwondo	Cerebral Palsy
Erwin	Male	Taekwondo	Brachial Plexus
Sandra	Female	Wheelchair Racer	Spinal Cord Injury
Jerry	Male	Wheelchair Tennis	Spinal Cord Injury
Nathan	Male	Wheelchair Tennis	Arthrogryposis
Frank	Male	Wheelchair Tennis	Spinal Cord Injury
Denise	Female	Wheelchair Tennis	Transverse Myelitis

Out of 29 participants, 18 were female and 11 were male. The nature of disabilities included physical, congenital, and acquired. Thirteen sports, both team and individual, were represented. The majority of the subjects had competed in the Paralympics prior and have qualified to compete in Tokyo 2020. While the majority had qualified, some were waiting on qualification and have not been given the chance to do so due to the disruption of COVID-19. Examples of sports represented included taekwondo, swimming, sitting volleyball, and goalball.

### Data Collection

Before data collection, an electronic confirmation was requested from the athletes upon review of the provided consent form. Twenty-nine in-depth, semi-structured interviews were completed with the duration of interviews lasting from 19 min to 1 h and 20 min. Data collection was conducted through virtual video calls under the university account to maintain confidentiality of participants. Twelve questions were sub-categorized into introduction/background, COVID-19 specific, and reflection-based questions (Table 2 provides sample interview questions).

**Table 2:** Sample interview Questions.

1. How did you get involved in the sport you are wanting to compete in?
2. How have the regulations affected your health regimen, such as your nutrition, training, and sleep?
3. Has your self-identity as an athlete been challenged during this pandemic?

All five research assistants were trained for the interview process by observing the tenured senior faculty member conduct two interviews; following, each researcher participated in a practice interview. Two researchers were present during data collection: a primary and secondary interviewer. The primary interviewer's role was to facilitate the flow of the interview and ensure all questions were addressed. The secondary interviewer primarily observed and provided follow-up questions when appropriate. A brief reflection by both researchers followed after data was collected. All interviews were recorded and transcribed.

### Data Analysis

Data analysis was conducted simultaneously with data collection and was employed using an inductive and comparative analysis strategy. Data was analyzed by the researchers using thematic analysis (Merriam, 2002) and a constant comparative method to maintain reliability and consistency. Organization of the analyzed data was inputted into an excel spreadsheet. First, three researchers individually analyzed one transcribed interview, then compared findings and determined a process for analyses. Each researcher was to be presented with 2/3rd of the data. This resulted in all transcripts being analyzed by two scholars, where they jointly discussed their analyses in a critical and constructive manner in order to refine the subthemes. By having two researchers analyze every interview, a level of accountability was shown throughout this process. Finally, all three researchers regrouped and agreed upon the final interpretation of the themes and categorized units.

## Findings and Discussion

### Prominent Strong AI, Multiplicity Over Exclusivity

The vast majority of the participants indicated that they strongly identified themselves as athletes at the time we interviewed them during COVID-19. Their strong AI originated from long-term commitment in sports, as well as benefits from involving and excelling in disability sport, including financial support, social opportunity, self-empowerment, and other life opportunities. By prioritizing the key athletic role and emphasizing the importance of sport while holding concerns of losing it, eight Paralympians stated they had exclusive AI. By contrast to exclusivity, the rest claimed that multiple roles in school, work and family accounted for their identities besides being an athlete. Interestingly, two athletes who underlined the multiplicity of their identities outside of sport, suggested that being an athlete was not a significant portion of their identities.

#### Overall Strong AI

When asked how much of their self-identity was rooted in being an athlete, 27 of the participants positively expressed a shared significant AI. This lends support to previous research (Groff and Zabriskie, 2006), which indicates that athletes with disabilities have equally strong AI compared to non-disabled athletes. Paralympians stressed its weight with different measurements by frequently quoting “a lot” “very strong” “almost all of it” “really big.” To support their statements, they automatically explained how much sports means to them and their lives through different lenses.

One crucial aspect of AI is the physical devotion to sport. Some of the athletes expressed enthusiasm and accumulated athletic experience in general sport participation. For instance, John emphasized that everything he “has ever done was sports-wise” while Campbell said he had an almost fixed athletic identity because “ever since I was little, I always tried to get into as many sports as I possibly could, and it's been like that ever since pretty much.”

With the maximized desire to improve and win, our participants focused on their goal-setting and devoted extensive efforts in training and competing, aside their fondness of sport. As Allie said, her strong AI was from committing to sport and then working as hard as needed. In addition, participants constantly mentioned the longevity of sport participation. As Erwin explained, “I spend the majority of my life training, working out, hanging out with my teammates and so yeah, I would say that's a good chunk, like probably, a large part of my identity.”

By prioritizing the athlete role, Paralympians planned life around sport and achieving sporting goals, but at the cost of the majority of their time and commitment. Maranda described her AI through the sacrifice of other activities or roles:

So that being an athlete is kind of my identity at the current moment because I have put so much time into it. And right now, it's where all my resources are going. It's planning around it. So, I've had to make a lot of sacrifices in the past 2 years to be able to make this dream a reality.

The confidence in their ability and their sense of self was unconditional, as Jerry indicated “before my injury and after my injury, tennis has always been a part of my life.”

Surprisingly, some of the athletes illustrated their strong AI by highlighting the situations when they were unable to compete. By going through injuries and similar experiences, Brittney related her AI to her strong determination to pursue excellence in volleyball. She claimed she would only quit until she couldn't play it “a hundred percent.” For John, He emphasized that even if he “was not competing in the Paralympics,” being an athlete will remain a huge part of his character. These experiences and reflections show that the athletes possess strong AI and have confidence in the stability of their AI, even throughout the COVID-19 pandemic.

Besides the impacts of long-term sport involvement to strong AI, athletes also underlined their appreciation for the physical and mental self-empowerment gained from disability sport. Sport not only provided them the opportunity to be active and healthy, but also offered other benefits that shaped who they are and changed their lives. The financial, psychological, and social benefits athletes received from sport, helped them easily identify their strong athletic persona.

Directly, some participants defined their strong AI regarding being an athlete as their occupation and livelihood, where sport provided them partial or full financial support. As Natalie pointed out, besides setting her goal to be the “greatest athlete that [she] can be,” having an athletic career was “also allowing [her] to financially be stable.” Moreover, while lack of social interaction and inclusion was a problem for many people with disability, our participants highlighted the independence and social opportunities they gained from the platform of sport and tournament participation. Participants were able to travel, meet people, interact with others and make friends. As Dennis recalled, being a Paralympian or just an adaptive athlete “has afforded me the opportunities to travel the world and to meet the friends I have.”

On the psychological side, through equal involvement and competition, sport allowed athletes to be competitive; build something concrete and be a better version of themselves, as Marge expressed:

The sport gave me an identity and I loved sports where things were really fair like jumping horses like the poles stay up or they come down, you know you go fast or you go faster, you know something concrete. I've always believed that you can try to be better at something and I think that identified me as an athlete.

Based on a concrete foundation, sport gave Paralympians identities outside of their disability, allowing them to self-identify as athletes, and be recognized as such by others. Having a master identity status was essential to them, as Brittney described:

That body image is huge, and it's been huge maybe like my whole life with my leg was always wanting to fit in or identify with something. It was very easy for me to identify with being an athlete. I look like an athlete, I act like athlete. I'm an athlete.

The self-definition from Brittney and other athletes illustrated that being an elite athlete provided a base to define and redefine themselves, think and behave beyond their disability. Moreover, the master role drawn from self-definition helped them to gain external recognition as athletes and resist stereotypes. Brittney further indicated when she was at school, “I'm the athlete like everyone knows that.” Being recognized and validated as athletes were important for them to reassure their identities. To Stewart, participating in sport or being a Paralympian could prove “that I'm deeper than just someone with a disability.” In Lily's experience as a Paralympian, she further identified as both an athlete and an advocate for disability sport and its benefits, and thus, “it's a gigantic part of” who she was.

More importantly, having a master identity as an athlete, along with strengthened competitiveness and confidence from winning and other achievements, the participants gained positive self-perceptions, which they have applied to other areas of life. Allie said, to “be on an equal playing field gave me a lot of confidence, and strengthened other areas of my life.” The positivity and achievements in sport also allowed Paralympians to pursue opportunities and lives outside of being an athlete, with enhanced self-esteem and other developed identities around sport (e.g., coach, advocate) or beyond sport (e.g., student, accountant). Thus, the multi-layer benefits of participation in adaptive sport were unanimously affirmed by Paralympians who commented: “there's so many layers” (Brittney); “it's so much more because it's just like ticks off all these boxes” (Natalie). Leslie elaborated on how sport shaped who she was and changed her life by offering so many opportunities:

Being an athlete has opened so many doors to opportunities, have influenced my life in such a positive way, through teaching opportunities through adaptive sports, and my life would be totally different without it.

In summary, Paralympians asserted that sport meant a lot to them because it influenced their lives in a greatly positive way, and they were grateful and proud of the outcome. As Stephanie stated, “I wouldn't have it any other way, you know, like it's made me into someone that I really proud.” The identity they brought into life and the experience learned from other events facilitated and reinforced their AI, as Leslie indicated: “I've learned like a lot of life lessons from being an elite level athlete and a lot of great qualities that I've carried into other experiences and vice versa.” The achievements in other roles constantly reminded them of the important roles derived from their athletic identities: “It's just like everything, like every little avenue that I've taken kind of steers back into my identity” (Natalie). We can see that being an elite athlete educated athletes in various ways, leading to progressions that would remind them of and reinforce their strong AI.

Indeed, through participation in disability sport and aspirations to win Paralympic medals, Paralympians developed self-acceptance and personal growth, along with the experiences of traveling and connecting with people around the world, leading to a purposeful life. These experiences were closely consistent with Taiwanese and British Paralympian participants' experience in previous research (Huang and Brittain, 2006), who suggested that sport participation and excellence gave participants a key identity as well as the ability to explore beyond their disability. However, in our study, instead of focusing on the impact of their disability status and transformative process, Paralympians were more positively focused on their strong athletic identity and multiplicity.

This study also confirmed contributors to stronger AI, such as devoting more hours to practice (Tasiemski and Brewer, 2011) and experiencing accomplishment and competence (Le Clair, 2011). It also supported the benefits associated with sports involvement and AI development, including perceived competence (Shapiro, 2003) and the ability to combat social marginalization and redefined as athletes by themselves and by society (Wheeler et al., 1999). Through this study, we further the discussion that strong AI allowed Paralympians to explore beyond athleticism to develop multiple identities through deeper interaction and engagement in society.

#### Exclusivity and Multiplicity

Based on the prominent strong AI, some of the athletes stressed their identities as being exclusively athletic, while more of the others stated alternative social roles and characteristics that complete the multiplicity of their identity.

Those who identified exclusively as athletes pronounced sport as a dominant anchor in life. Frank claimed: “Sports is my life, everything inside of me revolves around it. Sports is my world.” Athletes also highlighted the peak status of being a Paralympian, as Brandy states, “I'm at the height of my career right now. I mean, I qualified for the Paralympics, so you don't get any higher.” Due to age, Cara's first shot at the Paralympics would also be her last, so she felt her AI peaked at the moment as well. It seems like the exclusivity stemmed more from personal experience; nevertheless, these athletes shared the same appreciation of disability sport participation, on par with others expressing strong AI.

They had also described their participation in disability sport as a life-changing experience: “Man, without wheelchair sports I wouldn't be who I am today. It totally changed my life” (Kendall). Athletes were able to recognize the sole identity they were experiencing and the concern of losing that identity in the future:

It was so hard to stop and I've never really thought about that before because you know, I've always played sports and always played goalball. And so just to think about not playing anymore, I was like man, I … don't necessarily have an identity outside of goalball. (Connor)

While those who exclusively defined themselves as athletes struggled with finding identity outside of sport, there were comparatively more athletes in the study that claimed to intentionally plan for multiplicity or already have multiple identities. Yet for most of them, being an athlete was still their key identity. Jerry explained, “it doesn't take up my entire character.” It supports Martin (1999) found that many athletes' self-schemas are not limited solely to their AI. This is understandable because Paralympians, benefiting from confidence gained on court, often transition successfully to positions such as coaches, advocates, sport committee members, and other societal roles. Many of these identities came from sport and tie back to being an athlete. As Nathan reflected, “it's all connected back to playing the sport, but solely as an athlete probably 70% would be where I put my identity at and then you know, I definitely identify as a coach.”

Additionally, athletes described alternative social roles they held while engaging in society, whether familial, religious, or other roles. For instance, Maranda said, “I do have a career and I still find that as my identity as a working young professional also, as a family member.” Leslie detailed how she enjoyed her family role while concurrently an athlete: “I love being a parent. I love being a mom. I love being a wife.”

Both athletes who developed exclusive AI and multiple AI mentioned how sport as a foundation helped develop their identity. However, those with multiplicity consciously found other interests to balance and enjoy their lives, and purposefully planned out the post-athlete future to avoid exclusivity and its negative consequences. For instance, learning from stories of athletes experiencing deep loss with sport disruption, Jacob firmly believed that wheelchair rugby was a huge part of his life, but not the only part. He had to leave the sport for a time when injured, and this highlighted the need to learn life without sport, ensuring that upon retirement, he could say, “then I have a great life outside of sport.” In contrast, Michelle noticed her exclusivity while considering retirement, as she was constantly questioning herself: “Who am I without swimming? Who am I without a medal? What can I do and accomplish without swimming and a medal?” Not knowing their values without sport had forced some athletes to intentionally develop other identities, as Stewart added:

I'm more than just a swimmer. You know, I'm a good swimmer, but you know, I think it's important not to be all in on one thing. You need to have multiple outlets to pour your energy into, so to make yourself, you know, to not drive yourself crazy.

Paralympians realized that having identities outside of being an athlete is important for a well-balanced and diverse life. By their competitive nature and tendency to focus on a single activity, having multiple identities helps them maintain more balanced lives. As John shared:

So, training is my Yin to my Yang on balancing all the time that goes into work. It gives me something to keep my mind off work because I tend to get all consumed with whatever I'm going to do.

To further combat the pitfalls of exclusivity, we found that our participants actively planned for a future career, pursued higher education, and sought part-time employment even through retirement (in part due to financial concerns). There has been criticism for inequities in funding between Paralympic vs. Olympic athletes; the U.S. governing body provides smaller training stipends and pays smaller financial awards for medals win at a Paralympic Games. Among all the Paralympians in our study, only Stephanie identified herself as “a full time Paralympian right now” while the rest introduced themselves as athletes along with other roles. Leslie responded to her occupation as “a part time elite level athlete in the sport of goalball” and “also a part-time accounting assistant,” as she worked to support herself financially. It was worth noting that based on the strong AI, most of the athletes' priorities still remained in sport. However, other roles and identities could fulfill their lives when needed, as Katherine described:

“Being an athlete is something that I've always wanted to do and always strive for. But when it doesn't happen, then I have other things that I enjoy.”

However, with acknowledging that sports were critical to them, two Paralympians prioritized their multiplicity over their athletic identities and did not possess strong AI. It is necessary to discuss these two individual's cases, because it potentially resonates with the aforementioned discussions on the considerable time Paralympians devoted in major activities (e.g., education), the moment they reviewed their AI, and their plans for the future. For Valerie, even though “at this point, I feel like it's just eating up so much of my life fencing as an athlete,” she only identified a quarter of her identity as an athlete. We tend to believe that compared to her long-time work as a farmer, the short time she competed internationally and the time-consuming efforts she was making for graduate school during this time could be part of the explanation. Meanwhile, for Sandra, due to financial concerns, she chose to pursue graduate school. She stated that less than 50% of her identity was rooted in being an athlete because she stepped out of her exclusive athletic role years ago for a more certain future:

I'm starting to think about what's coming after my athletic career. Yeah, and so I think in 2017 just because of grad school and realizing that it's not very sustainable to be relying on like U.S. Paralympics for my health care and like my health insurance just because like you have to almost get a world record every year to keep your health insurance. And so, it just became more and more apparent that I'm like, okay. Logically, I can't do that forever.

We conclude that Paralympians were having prominent strong AI during COVID-19. The overall strong AI and partially exclusive AI in Paralympians supported that elite athletes have stronger AI (Tasiemski et al., 2004). As we explored the paths of Paralympic hopefuls, we found that their strong identities were consistent with what they described as good experiences of participating in disability sport and all the benefits accompanying it. These factors work as facilitators to help with strong AI development and maintenance. One prominent facilitator observed is how Paralympians justify the importance of the Paralympics to the athletes.

In our study, AI development facilitated athletes' positive personal and social identities which supported the affirmative mode. Consistent with previous studies, Paralympians use sport participation as a means to repair and redefine self-identity, increase sense of pride, normalize physical appearances to the greater society, and catalyze personal empowerment in non-sport contexts (Swartz et al., 2019). Yet our athletes fully embraced their disability status along with their athlete status. Thus, throughout the current study, the notion of multiple identities was not narrowly based on the discussion of the negotiation process between a disability identity and athletic identity, but more emphasized on the meaningful identities developed beyond it.

We understand individuals can draw their self-identity from a variety of sources. In the current study, most of the Paralympians established their identity by their primary time-consuming activity (e.g., sport, occupation). However, we should also notice that even with the same level of commitment, efforts, and achievements, there were athletes that did not express a strong AI. According to previous research, we would assume that our participants, who have devoted intensive efforts and have great achievements through professional mega disability sporting events, would tend to have more exclusive AI. Yet, through a qualitative manner and under each participant's individualized experience, we are able to identify that more Paralympic Hopefuls express identity multiplicity over exclusivity. When participants identified themselves through other roles other than their athletic role, they did not possess a strong AI. It is a unique phenomenon compared to their non-disabled counterparts (e.g., Olympian), who tend to have a one-dimensional athletic identity.

We clearly observed the multiplicity and fluidity aspects of identity construction through our participants. It seems that athletes realized sport “is not everything” or should not be the only defining identity due to the impact of exclusivity, funding issues, and career considerations. These critical factors compelled them to make conscious decisions to commit to other activities and develop identity multiplicity, and were not identified by previous research. According to Wheeler et al. (1999), active elite athletes with disabilities seemed not to consider or think about their future life outside of sports, which might cause future transitional problems. With acknowledging their considerations, the governing body should accordingly provide career consultation, more funding opportunities, and other environmental support to help athletes develop and maintain their strong AI.

Also noting that by the time the worldwide COVID-19 pandemic took place, their identities were shifting due to their life situations. This supports Huang and Brittain's (2006) study observing that identities shift over time as people change, and we will illustrate this theme more thoroughly through the following section.

### Various Impacts on AI, Mental Adaptation Helps Overcome the Lack of Sport Participation

Given the status of Paralympian's AI, we investigated the effects of the pandemic sport disruption and whether the athlete's AI were challenged. Three major subthemes emerged from our data, with athlete's AI being: (a) Challenged, (b) Unchanged, and (c) Strengthened. We hope this section sheds light and gives a voice to not just elite athletes, but the unique population of Paralympians. Given that there can be such harmful repercussions from sport disruptions on athlete's AI, the lived experiences discussed from this study are beneficial to sport personnel and governing bodies in assisting elite athlete's more adequately with their struggles.

#### Challenged AI

When athletes were asked about the COVID-19-related impacts on their AI, a slight majority of our Paralympians described their AI as being challenged and negatively impacted. The pandemic caused these athletes to feel as if their AI had been completely lost, and they struggled psychologically, feeling that they could no longer define themselves as “athletes.” The most prominent reason for these AI challenges was the strong tie between AI and the physical aspect of sport. With facility closures, cancellations of competitions, and overall decrease of sport participation due to COVID-19, the athletes' AI faltered along with their lack of athletic activity. It was difficult for them to identify as athletes without the continuous training and high-level competition they practiced prior to the pandemic. This relationship is explained by Abbie, who experienced a decrease in training that resulted in her AI being challenged:

You know, it's kind of been a little more challenging, I guess. Because then you know, if you can't train then you don't necessarily represent being an athlete, you know. It's like, 'oh I worked out like three times in the past month' and that's not really being an athlete. It's actually really sad. So, I've kind of been a little stir-crazy not being able to train.

Allie also describes how she struggles to have an athletic identity without participating in her sport. The long period unable to play causes her to question her athlete status.

We honestly haven't competed since January. That is insane for us, like that's 6 months. So, it's like I know I need to be back out there and compete and I know, you know being an athlete is a big part of my identity, but I kind of just feel lost right now.

The identity loss experience by Allie is shared by among all of our athletes in this subtheme. For these athletes sport was not just activity they took part in, but it is how they defined themselves. Defined by the title “Paralympian,” they felt the need to prove themselves in training and competing on the high-stage of Paralympics. With the event's postponement athlete's no longer had this title to identify with, and questioned themselves as Maranda did:

It has definitely been challenged because I haven't been able to get on the court. I haven't been able to prove myself as an athlete and like I said before it was kind of like well the Paralympics don't happen then who am I now?

Coupling this loss of identity, our findings revealed that “Challenged AI” athlete's experienced negative impacts on their mental health. The decision to postpone the Paralympics a year was hard to cope with for these athletes, as many of them prepared intensely in the 4 years leading up to the event. Reflecting on their current lifestyle revealed how different it was prior to the pandemic. Natalie goes on to describe how the lack for sport has affected her mental health:

I think the only thing that it did mess with was my mental because you know, you're so used to a lifestyle. I'm so used to always being on the go, training and traveling and I used to fly at least once a month and now it's nothing, like I don't go anywhere.

This loss of identity and identity confusion due to sports disruption are consistent with what was observed by Caron et al. and Kerr and Dacyshyn (2017; 2000) when studying identity effects from injury and retirement. Because injury and retirement lead to a complete or partial stop of sport participation, the athletes' mental health suffered severely. These challenges are not only caused by loss of identity and of sport, but from the disruptions in other areas of their lives. It alters their life purpose, social network, and overall lifestyles in a negative way, resulting in symptoms of depression and anxiety. With these factors and much of their AI revolving around their sport, the sudden halt forced athletes into an uncomfortable and stressful situation. Similarly, Muscat's (2010) findings revealed that elite athletes struggled to adapt to a life without sport, one that is not structured by training, coaching, and competition. Further, the abrupt nature of the lockdown and facility closure intensified the reaction by athletes, as there was little anticipation- not allowing time for athletes to cope and adjust. Wippert and Wippert (2010) discuss how the involuntary exiting of sport has a significant impact on psychopathological symptoms, with a higher prevalence for depression, anger, and hostility.

#### Unchanged AI

An almost equal portion of athletes did not feel their AI had been challenged. In contrast with the participants who experienced a challenged AI, these athletes' AI consisted of more than the physical aspect of sport. Athletes describe how they have internalized the sport and maintain the self-image of an athlete, so much so that their AI is unwavering despite decreased activity:

I have so much confidence in myself. It doesn't matter if I haven't practiced in 10 years. I'm still going to go out there and beat everybody, that's just the self-confidence…I just go to strive to be the best at what I can do. It doesn't matter if I'll practice or not. At the end of all this (the pandemic), I'm going to go out there and play. I'm giving my whole heart, mind, body, and soul to whatever the outcome is. That's what's going to happen here. That's what I have to do. (Frank)

For Frank the relationship with sport is much more than being physically active, it carries an existential tie and meaning for him. Frank describes how sport is at the center of everything he does, and even with the delay of playing he feels obligated to continue his purpose to be the greatest athlete he can be.

This kind of confidence and athletic manifestation is further expressed by Valerie. For her, being an athlete is not simply an activity she participates in, but it is how she identifies regardless of the situation she is in. The pandemic seems to be a period where she is needing to change the way she completes tasks, but does not alter her self-perception:

So, like I'm pretty secure in who I am as a person. So, like if I can't figure it out, like how to be active at home that's on me. I stay active where I can and even if I'm not being active and being lazy for a while, I know I'm still an athlete. I can still do sports. Just because I'm not doing it now doesn't mean I'm not an athlete.

These athletes were very firm in their idea of self and were able to separate their emotional distress and their AI. Brandy, explains how her mental health suffered without her standard training structure. She states that although she went through a period of depression, her AI remains unchanged:

I would definitely say I went through a minor like depression phase I think just because I'm so used to routine and not having a routine like definitely stressed me out. I wouldn't say I question myself as an athlete. At the end of the day, I'm still an athlete, quarantine, no quarantine, Corona no Corona, like I'm still an athlete. I'm confident in that aspect, but it was just like I thrown off of my routine and stuff like that.

These athlete's perspectives speak to this groups' confidence and positive mentality maintained throughout the pandemic. Their self-concept as an athlete remains intact as they focused on the pandemic as a temporary event rather than changing their commitment to their sport and the Paralympics. Describing these athletes as focused, emotionally intelligent, and adaptable, they are in line with the Paralympians' “mental toughness” characteristics discussed by Powell and Myers (2017). Paralympian's mental toughness is conceptualized from specific cognitive strategies (rational thinking and goal setting), cognitions (connection and non-acceptance of constraints), and specific characteristics including optimism, pragmatism, resilience, and self-belief (Powell and Myers, 2017). The observation of AI being unaffected by sport disruption is one that is not expounded on much in the related body of literature. In previous research, much of the discussion on sport disruptions and AI revolves around the struggles athletes face, many times resulting in negative impacts on their identity. With these participants, however, we recognize and discuss how Paralympians' AI remains fixed and salient, in spite of a pandemic.

An interesting perspective shared by a few of the athletes across challenged and unchanged AI, was that they viewed the pandemic as a way to break from their strict AI, giving them an opportunity to explore other activities. Instead of feeling anxious and uncertain about not being able to participate in their sport, the break was a period to figure out how to navigate life without sport. A test for retirement is how athletes described it. For Nathan, sport was a significant part of his life from an early age. He explains how thoughts of life without sport worried him and made him question whether his mental health would be sound once he retired. Anticipating this internal conflict, he decided to take advantage of this time off to prepare himself mentally.

Here's the one interesting thing with COVID. It was somewhat of a preview of what my life is going to be after I retire, because all of a sudden, I'm not traveling and not going to compete or play tennis tournaments. To be honest, I mean, it's been my life since I was 14, so I was scared. I don't know how much you guys have read about athletes who have been athletes their whole life and all of a sudden when they stop it's scary. They don't know what they could do themselves, and I knew that I was going to be that way. So, this has kind of been a test run for that. So, I think that mentally it shed light on what it's going to be like and I think it's given me some good ideas of things to do when I quit so I don't, you know, go into some mental dark place and stuff like that.

Lally (2007) has observed that loss of sport can preclude AI challenges, if approached the correct way. This can be achieved by in-depth thought and consideration for what life is like after sport, and this anticipation can be beneficial in coping when departing from sport, much like in Nathan's discussion. Anxiety over exclusive athletic identity in the face of retirement or sport disruption is common in the elite athlete world. Athletes often realize that without other meaningful identities outside of sport, feelings of emptiness may result when sport is lost. The importance of developing identity in other avenues has been expressed by prior research (Kerr and Dacyshyn, 2000; Muscat, 2010), stating that competing at an elite level of sport can be detrimental if there is no effort to maintain a balance in other life activities. Loss of identity can result from athletes lacking the confidence to explore new areas (Muscat, 2010). That being said, for the athletes seeking more identifiers beyond their AI, they consciously acknowledged the importance of identity multiplicity.

#### Strengthened AI

Three athletes with both “challenged” and “unchallenged” AI explicitly state that they feel their AI has been ultimately strengthened. As athletes learned to develop other fulfilling roles during the pandemic, the hiatus from sport interestingly strengthened their AI. Kendall explains that after 16 years of a sport-dominated lifestyle, she embraced other interests:

I'm not training at a Paralympic level right now that I would normally be leading up to the games. I think it's (AI) strengthened because I found acceptance. I found peace in myself doing other things that make me happy and being active like I've really gotten into hiking a lot and biking like I know that like I can do other activities and they still make me feel good inside and maybe I don't need to be at that like super high level or on that stage all the time. I think I've never had that because I've always been training for a “games” or something. So, in this, I really think it has strengthened my identity as an athlete because I'm okay that I don't have to be amazing at something. I can suck at something and I'm okay with it now, whereas before it was a lot harder to accept.

In contrast to what “Challenged AI” athletes stated about their identity loss from being away from sport, it seems that “Strengthened AI” athletes express that they had found identity within the break. Without the pressures from their sport- training routine, coaching, and competition they gain confidence within themselves and their identity as a whole. Marge, an equestrian athlete, states how she felt a sense of freedom from her athletic lifestyle, even though she enjoys being an athlete. This allowed her to gain confidence in her identity without external pressures. When asked if she felt her AI has strengthened, she states:

Yes. Yes, I do because it builds resourcefulness and a lot of times especially with our sport. There's a lot of coaches involved and honestly, for lack of a better phrase, a lot of egos and power trips and control. Right now, the only person that can really control me is myself, and so that's reinstalled confidence.

She further describes her AI in relation to her sense of liberation:

Yeah I still have value as an athlete. I still am motivating myself to do the best I can and now it's not like I have somebody sitting there telling me what to do every 2 s. I have a certain freedom and can make choices for myself and for my horse as well. He's never seemed happier, you know, so sometimes it's kind of good to go back to your roots, connecting with what you love about the sport and you still have your hopes and goals and dreams, you know, it's just a different way of doing it.

For Brittney, she developed a new sense of AI. She speaks as if before the pandemic she had been taking for granted the ability to connect with her teammates, and practice. Because of the halt in her sport participation, she feels a sense of loss, however it seems she subconsciously knows that this break is short-term, thus making her want to participate in her sport more than before. She gains appreciation and a newfound admiration for the sport she no longer could participate in. Until the pandemic, she had not taken the time to think on why she continues in her sport. Brittney questions:

I also feel like it makes me enjoy playing volleyball even more. Is that weird? I appreciate when I go out and play even though it's not like I can play with anyone. Like there's a reason why I started playing volleyball like I love it. So like these things that I've not necessarily embraced before have given me this platform and showed me this is what sent me on this path to really identifying as an athlete again.

For Brittney and Marge, the cessation from sport allowed them to reflect on the state of their AI before the pandemic, and realize it was not the same after years within their athletic career. Without their focus on competition, training, and coaching they re-centered their AI, and ultimately felt more enjoyment and confidence in their identity. Kendall, however, feels more comfortable in her AI by adding more aspects to her identity wholistically. She also spoke on her dedication to training and how it is, in some ways, detrimental to her mental health- with the constant desire to perform at a high level. Further describing her growth in finding acceptance when she cannot meet such high standards.

The observation of AI strengthening from developing identity outside of sport, is a relationship that is not common in the body of literature involving sport disruption. Even so, sport disruption resulting in AI strengthen is most similar to that of the sport disruption of motherhood. Because our 'Strengthened AI' athletes emphasize the development in their AI, similarities can be drawn from their growth and the enlightenment to that observed in mother-athletes. Prior to motherhood, pregnant elite athletes maintained their strong AI with much of their whole identity constructed around sport (Tekavc et al., 2020). Following childbirth, these athletes developed a dual identity, and were observed to experience a stronger motivation to pursue their athletic goals and an overall stronger desire to continue engagement in their sport (Tekavc et al., 2020). This reaction to sport disruption is similar to what was discussed by Brittney and Marge, as their motivation and passion grew for their sport. Further on motherhood, elite athletes' self-identity has also been seen to benefit from identity multiplicity by reducing the competitive pressure of sport (Appleby and Fisher, 2009). This relationship of AI benefitting from sport disruption is described by our participants Kendall and Marge, as they thrived without the strict structure of their athletic lifestyle. As for the reduced sport pressures strengthening AI, this perspective is inconsistent with other studies where athletes have been shown to feel uncertainty about their future lives without the structure and routine sport gives them (Kerr and Dacyshyn, 2000; Menke and Germany, 2019).

In summary, athletes who experienced AI challenges in COVID-19 did so as a result of not being able to participate in sport; focusing on the temporary loss of their sport. This progressed to identity loss and ultimately symptoms of depression and anxiety. Speaking to the “unchanged” and “strengthened” AI athletes perspective, though experiencing a similar situation and lifestyle change as the challenge athletes, they seemed to develop a mental adaptation and create a positive outlook as a way to cope with the sport disruption. Instead of dwelling on the negative emotion felt by the postponement and the decreased sport participation, they chose to use the situation as an opportunity to self-explore. In addition, in documenting these athletes' perspectives and the reasoning for them, we hope sport management organizations heed them to create a more supportive system- accommodating to AI difficulties through sport disruptions.

## Conclusion

Participants in this study possessed prominent strong athletic identities. Rather than identifying with just their athletic role, athletes self-identities were comprised of multiple responsibilities and activities during COVID-19. We found there were about equal numbers of participants who claimed that their AI has been predominantly challenged, unchallenged, and who felt their identity was both unchanged, strengthened and challenged. Findings indicated that having strong AI did not necessarily result in negative identity impacted by the sport disruption of COVID-19. In addition, COVID-19 did not challenge those who do not possess a strong AI.

Paralympians who developed multiple roles in life mostly felt that their AI has been negatively challenged. This is likely because of their singular focus on the loss of physical participation in sport during COVID-19. These Paralympians strongly structured their identity in their physical devotion to being an athlete. In contrast, athletes who have exclusive AI tended to report the mixed feeling of identity change due to the sport disruption of COVID-19. The reduced sport participation definite impacted their AI, yet they reported having the confidence and security in their AI. For participants who were able to adapt to the situation and maintain a stable AI (e.g., AI was unchallenged or even strengthened) during COVID-19, the mental preparation and adaptation was consistent.

Some of these athletes treated the situation resulting from COVID-19 as a test run for retirement and an opportunity to pursue other interests. Other Paralympians developed appreciation for their sport by keeping their athletic mindset, continuing with their previous training commitment. These athletes shared a positive outlook on the COVID-19 pandemic in relation to their AI. In conclusion, Paralympians' mindset of how to view and interpret their AI is crucial to how the individual's AI is affected by COVID-19.

We believe this study is the first of its kind, detailing how Paralympic athletes' identities have been impacted by the unique sport disruption cause by COVID-19 pandemic. In terms of theoretical implication, our study adds to the body of knowledge regarding social identity, athletes with disability in general, and Paralympians- a group that literature has not focused on. The findings of the study prepared for further discussion of disability sport AI through a quantitative lens. In addition, this study provides a clearer picture as to how and why athletic identities are impacted by career transitions. We believe that this research will provide sport governing bodies, coaches, athletes, and other sport personnel with a better understanding of the impacts of major athletic disruptions and how to provide more effective support for these identity-challenging events. Sport managers should work on creating environmental support to provide athletes more time for quality training to maintain their strong AI. Consultation service and funding opportunities needs to be provided by sport managers to help Paralympians avoid exclusivity and develop multiple identity for post-athlete life transition. Specifically, administrators could assist athletes transfer sport skills to daily life situations and career planning. Thus, to help Paralympians decrease their AI by investing in other roles for their post-athletic lives.

### Limitations and Future Research

The present study is not without limitations, but also presents important opportunities for future research. This study represents disability sports only in the United States. Funding and recognition differ in other countries and organizations (Hums et al., 2003). Cottingham et al. (2015) note that the vast majority of research in disability sport focuses on western contexts.

Based on this limitation, we would encourage future researchers to examine National Governing Bodies (NGBs) in differing geographical areas and their response to the effects of COVID-19, particularly 6 months after the 2020 Paralympic Games, as the impacts and implications of COVID-19 seem to go well-beyond the games. Implementation of new regulations made by governing bodies can be tested to see if they are beneficial, sustainable in time, and advantageous regardless of the pandemic. Responses made by NGBs that addressed the training of the athletes and their sport participation can play a part in the formation or break down of an athlete's self-identity. Furthermore, these new regulations may be considered as a sport disruptor to the way these individuals train. Athletes already fight for more representation and view the Paralympics and being a Paralympian as a career. Guidelines like no spectators, that can lead to less promotion, which then leads to less sponsorship—should be examined to see if a correlation between these factors can further affect an athlete's identity.

Another limitation of the study is the duration of time the interviews took place. Interviews were conducted during the months of July and August 2020, only capturing a narrow window of time during the pandemic. The ongoing pandemic can further influence training and the mental and physical state of athletes for months or years to come.

This study focuses on the identity of athletes where disruptors from the pandemic subsequently caused a shift in their self-perceptions of identity. It is possible for athletes' identity and experiences to change after the interviews. Future research could focus on quantifying the self-identity change with Athletic Identity Measurement Scale to generalize the sample and examine a subject's AI using a quantitative method. The construct of an individual's identity needs to be better represented, because the measurement of identity is never identical. The exploration of identity formation and experiences in athletes are few, resulting in many questions remaining on this topic (Muscat, 2010). Various factors lead to identity formation and continue to develop new roles in an individual. As identities are constantly in the process of change and transformation (Hall and Du Gay, 1996), follow up research is needed to explore how athlete identities evolve.

## Data Availability Statement

The raw data supporting the conclusions of this article will be made available by the authors, without undue reservation.

## Ethics Statement

The studies involving human participants were reviewed and approved by Institutional Review Board of the University of Houston. The patients/participants provided their written informed consent to participate in this study.

## Author Contributions

TH contributions to this study involved reviewing and analyzing the literature body of athletic identity (AI) and its significance to Paralympians, and also wrote about the prominence of AI in Paralympians in the results section. In addition, TH contributed to the abstract, introduction, and conclusion parts of the paper. MM contributions included reviewing and analyzing the literature body surrounding sport disruptions and wrote about the impacts (challenged, unchallenged, and strengthened) on AI. JC contribution included writing about the methods incorporated in this study, as well as the future research suggested to expand the literature body on AI. MC contributions involved providing guidance and overlooking research assistants work throughout the study. All authors contributed to the article and approved the submitted version.

## Conflict of Interest

The authors declare that the research was conducted in the absence of any commercial or financial relationships that could be construed as a potential conflict of interest.
